# Supplementary material for: Sliding of HIV-1 reverse transcriptase over DNA creates a transient P pocket – targeting P-pocket by fragment screening
Source: Nat Commun. 2021 Dec 8;12:7127. doi: 10.1038/s41467-021-27409-y (PMC8654897; doi:10.1038/s41467-021-27409-y)
Supplement: Supplementary file 1 — Supplementary Information [file 41467_2021_27409_MOESM1_ESM.pdf]

Supplementary Materials

Sliding of HIV-1 reverse transcriptase over DNA creates a transient pocket – Targeting P-pocket by fragment screening

Abhimanyu K. Singh,[a,1] Sergio E. Martinez,[a,1] Weijie Gu,[a,b] Hoai Nguyen,[b] Dominique Schols,[a] Piet Herdewijn,[b] Steven De Jonghe,[a] Kalyan Das[a,*]

[a]Department of Microbiology, Immunology and Transplantation, Laboratory of Virology and Chemotherapy, Rega Institute for Medical Research, KU Leuven, Herestraat 49, 3000 Leuven, Belgium

[b] Department of Pharmaceutical and Pharmacological Sciences, Laboratory of Medicinal Chemistry, Rega Institute for Medical Research, KU Leuven, Herestraat 49, 3000 Leuven, Belgium

[1]Contributed equally.

*Correspondence: kalyan.das@kuleuven.be

Contents

Supplementary Table 1. X-ray crystallography data collection and refinement 3

Supplementary Fig. 1. Crystal symmetry interaction creates P-pocket 4

Supplementary Fig. 2. A schematic overview of the experimental setups used in XChem facility to perform fragment screening by X-ray crystallography 5

Supplementary Table 2. Virtually designed fragments 6

Supplementary Table 3. Docking results and drug-like properties of hits and selected fragments 7

Supplementary Fig. 3. Docking mode and detailed interactions of designed fragments F01-F05 with HIV-1 RT/dsDNA 8

Supplementary Fig. 4. Interactions of (a) **F47** and (b) **F81** with HIV-1 RT/dsDNA 9

Supplementary Fig. 5. Size-exclusion chromatography of HIV-1 RT/37-mer hairpin-DNA aptamer complex 10

Supplementary Table 4. Single particle cryo-EM data and structure analysis statistics 11

Supplementary Fig. 6. Impacts of crystallography and cryo-EM experimental conditions on the complexes 12

Supplementary Fig. 7. Cryo-EM density maps for **166** and **F04** in stereo 13

Supplementary Fig. 8. Superposition of RT/dsRNA structures with apo RT/DNA P-1 complex structure 14

Supplementary Fig. 9. Cryo-EM data processing 15

Supplementary Fig. 10. RT inhibition assay supporting figures 16

Supplementary Methods

- Fragment design and docking study 17
- Synthesis of fragments (Supplementary Figures 10 - 13) 18

NMR spectra and HRMS of fragments **F01 – F05** 22

References 30

Supplementary Table 1. X-ray crystallography data collection and refinement statistics.

**Data collection**	**Apo RT/DNA**	**Fragment 048**	**Fragment 166**
Synchrotron Beamline	Diamond I04	Diamond I04-1	Diamond I04-1
Wavelength (Å)	0.91587	0.91589	0.91589
Space group	*C*2	*C*2	*C*2
Molecule/a.s.u.	2	2	2
Cell dimensions			
a, *b*, *c* (Å)	310.63, 62.07, 168.23	309.57, 61.90, 168.78	310.74, 62.06, 169.29
α, β, γ (°)	90, 104.51, 90	90, 104.55, 90	90, 104.93, 90
Resolution, Å	81.44 – 2.85	98.75 – 3.30	150.13 – 3.37
(highest resolution shell)	(2.91 – 2.85)*	(3.42 – 3.30)	(3.43 – 3.37)
Unique reflections	73287 (4471)	44516 (4374)	44790 (2220)
R_{merge}	0.234 (1.413)	0.323 (0.953)	0.329 (2.88)
I / σ(I)	5.9 (1.5)	3.3 (1.4)	3.4 (0.5)
$CC_{1/2}$	0.991 (0.243)	0.973 (0.251)	0.987 (0.338)
Completeness (%)	99.8 (100)	94.6 (95.3)	99.9 (100)
Redundancy	6.2 (6.2)	3.4 (3.5)	5.7 (5.9)
**Refinement**			
Resolution (Å)	2.85	3.30	3.37
R_{work} / R_{free}	0.21/0.24	0.26/0.29	0.22/0.26
No. atoms	17865	17578	17725
Macromolecules	17669	17530	17613
Ligand[#]	-	26	29
Water	157	-	19
B-factors (Å^2)			
Macromolecules	63.86	63.27	98.62
Ligand	-	77.39	121.49
Water	44.73	-	73.50
R.m.s. deviations			
Bond lengths (Å)	0.003	0.005	0.005
Bond angles (°)	0.550	0.905	0.862
Ramachandran plot			
Favoured/allowed/ outlier (%)	96.97/2.87/0.16	96.56/3.18/0.26	96.87/2.97/0.16
Rotamer			
Favoured/poor (%)	80.99/0.75	91.88/0.29	91.49/0.17
MolProbity scores			
Protein geometry	1.55 (100th)	1.90 (100th)	1.84 (100th)
Clash score all atoms	6.86 (100th)	16.44 (97th)	13.96 (97th)
PDB code	7OZ2	7OXQ	7OZ5

*values in parentheses are for highest-resolution shell.

[#]bound fragment.

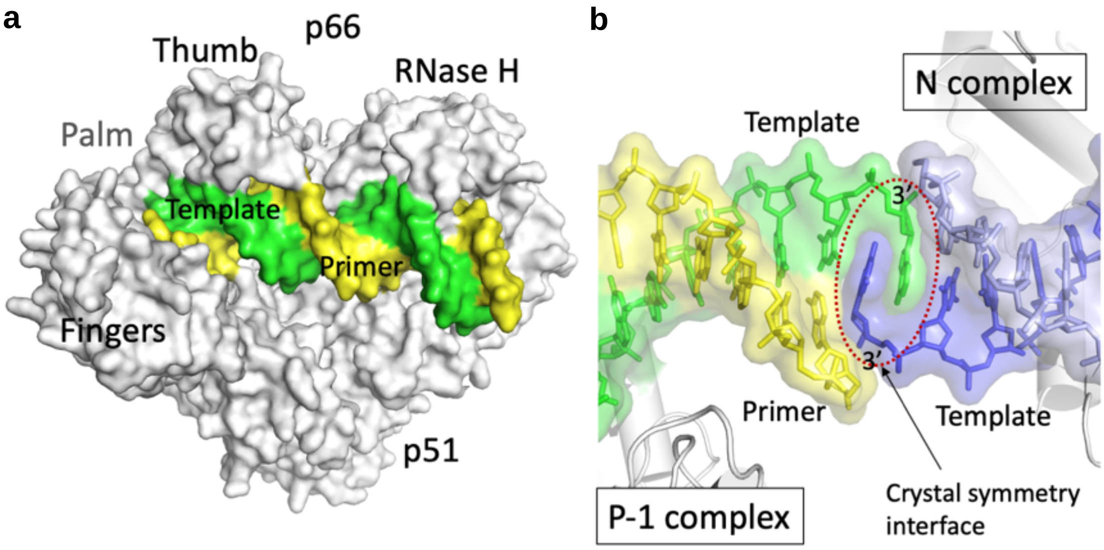

Supplementary Fig. 1. Crystal symmetry interaction creates P-pocket. a Space-filling model of RT (gray) with dsDNA (yellow primer, green template). **b** Crystal symmetry interaction between the DNA duplexes of P-1 complex (left; green and yellow) and N complex (right; dark and light blue) stabilizes the P-1 complex with a transient P-pocket in crystal.

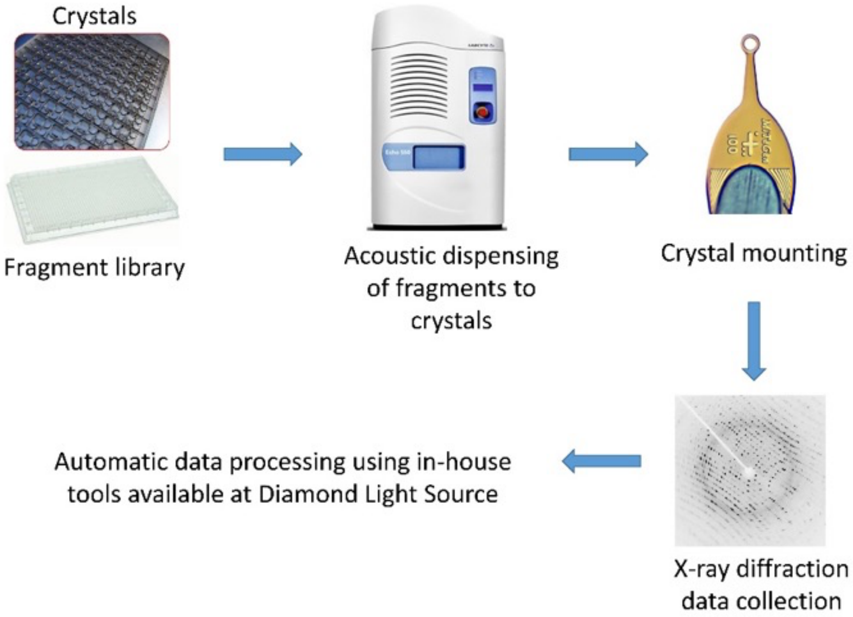

Supplementary Fig. 2. A schematic overview of the experimental setups used at XChem facility for fragment screening by X-ray crystallography. Fragment libraries were stored in 96 well plates; one fragment solution per well. An acoustic dispenser was used to dispense a small volume of a given fragment directly to a drop on a 96-well crystallization plate containing crystals in each well. After soaking, one crystal per fragment were mounted on cryogenic loops[4] flash frozen in liquid N_2 and loaded to the I04-1 beamline for automatic data collection.

Supplementary Table 2. **Virtually designed fragments.**

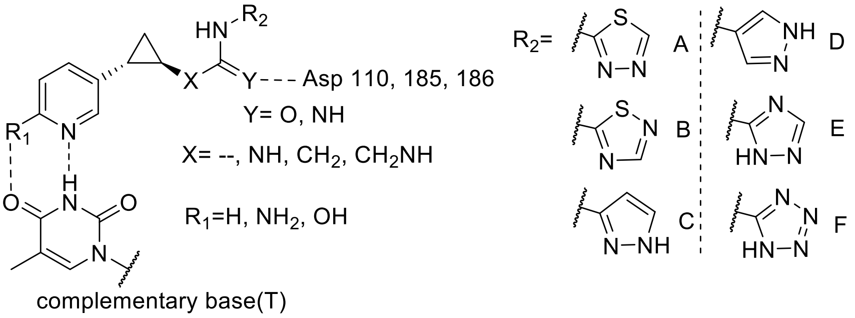

Frag.	R_1	R_2	X	Y	Frag.	R_1	R_2	X	Y	Frag.	R_1	R_2	X	Y
F01	H	A	--	O	F29	NH_2	E	--	NH	F57	H	C	CH_2	NH
F02	H	B	--	O	F30	NH_2	F	--	NH	F58	H	D	CH_2	NH
F03	H	C	--	O	F31	OH	A	--	NH	F59	H	E	CH_2	NH
F04	H	D	--	O	F32	OH	B	--	NH	F60	H	F	CH_2	NH
F05	H	E	--	O	F33	OH	C	--	NH	F61	NH_2	A	CH_2	NH
F06	H	F	--	O	F34	OH	D	--	NH	F62	NH_2	B	CH_2	NH
F07	NH_2	A	--	O	F35	OH	E	--	NH	F63	NH_2	C	CH_2	NH
F08	NH_2	B	--	O	F36	OH	F	--	NH	F64	NH_2	D	CH_2	NH
F09	NH_2	C	--	O	F37	H	A	NH	NH	F65	NH_2	E	CH_2	NH
F10	NH_2	D	--	O	F38	H	B	NH	NH	F66	NH_2	F	CH_2	NH
F11	NH_2	E	--	O	F39	H	C	NH	NH	F67	OH	A	CH_2	NH
F12	NH_2	F	--	O	F40	H	D	NH	NH	F68	OH	B	CH_2	NH
F13	OH	A	--	O	F41	H	E	NH	NH	F69	OH	C	CH_2	NH
F14	OH	B	--	O	F42	H	F	NH	NH	F70	OH	D	CH_2	NH
F15	OH	C	--	O	F43	NH_2	A	NH	NH	F71	OH	E	CH_2	NH
F16	OH	D	--	O	F44	NH_2	B	NH	NH	F72	OH	F	CH_2	NH
F17	OH	E	--	O	F45	NH_2	C	NH	NH	F73	H	A	CH_2NH	NH
F18	OH	F	--	O	F46	NH_2	D	NH	NH	F74	H	B	CH_2NH	NH
F19	H	A	--	NH	F47	NH_2	E	NH	NH	F75	H	C	CH_2NH	NH
F20	H	B	--	NH	F48	NH_2	F	NH	NH	F76	H	D	CH_2NH	NH
F21	H	C	--	NH	F49	OH	A	NH	NH	F77	H	E	CH_2NH	NH
F22	H	D	--	NH	F50	OH	B	NH	NH	F78	H	F	CH_2NH	NH
F23	H	E	--	NH	F51	OH	C	NH	NH	F79	NH_2	A	CH_2NH	NH
F24	H	F	--	NH	F52	OH	D	NH	NH	F80	NH_2	B	CH_2NH	NH
F25	NH_2	A	--	NH	F53	OH	E	NH	NH	F81	NH_2	C	CH_2NH	NH
F26	NH_2	B	--	NH	F54	OH	F	NH	NH	F82	NH_2	D	CH_2NH	NH
F27	NH_2	C	--	NH	F55	H	A	CH_2	NH	F83	NH_2	E	CH_2NH	NH
F28	NH_2	D	--	NH	F56	H	B	CH_2	NH	F84	NH_2	F	CH_2NH	NH

Supplementary Table 3. Docking results and drug-like properties of hits and selected fragments.

Fragment No.	Chemical structure	Affinity (kcal/mol)	Number of H bond interactions[a]	M.W.	cLogP[b]	Interacting surface area ($Å^2$) of ligand[c]
048	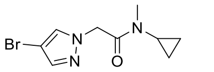	-5.1	1	258.12	1.07	333.3
166	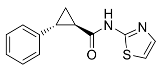	-5.9	2	244.31	2.68	339.5
F01	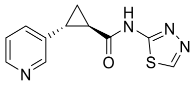	-6.7	0	246.29	0.86	347.8
F02	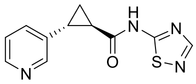	-6.2	1	246.29	0.87	332.8
F03	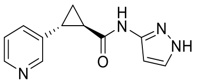	-6.7	2	228.26	0.35	348.4
F04	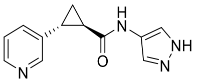	-6.7	2	228.26	0.63	367.7
F05	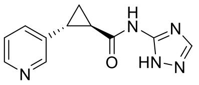	-6.4	2	229.24	0.31	377.2
F47	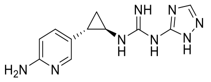	-6.6	3	258.29	-0.12	378.9
F81	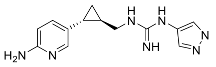	-7.6	4	271.33	0.78	438.8

[a] Calculated from LigPlot.

[b] Calculated from http://www.vcclab.org/lab/alogps/.

[c] Generated from https://www.ebi.ac.uk/pdbe/pisa/

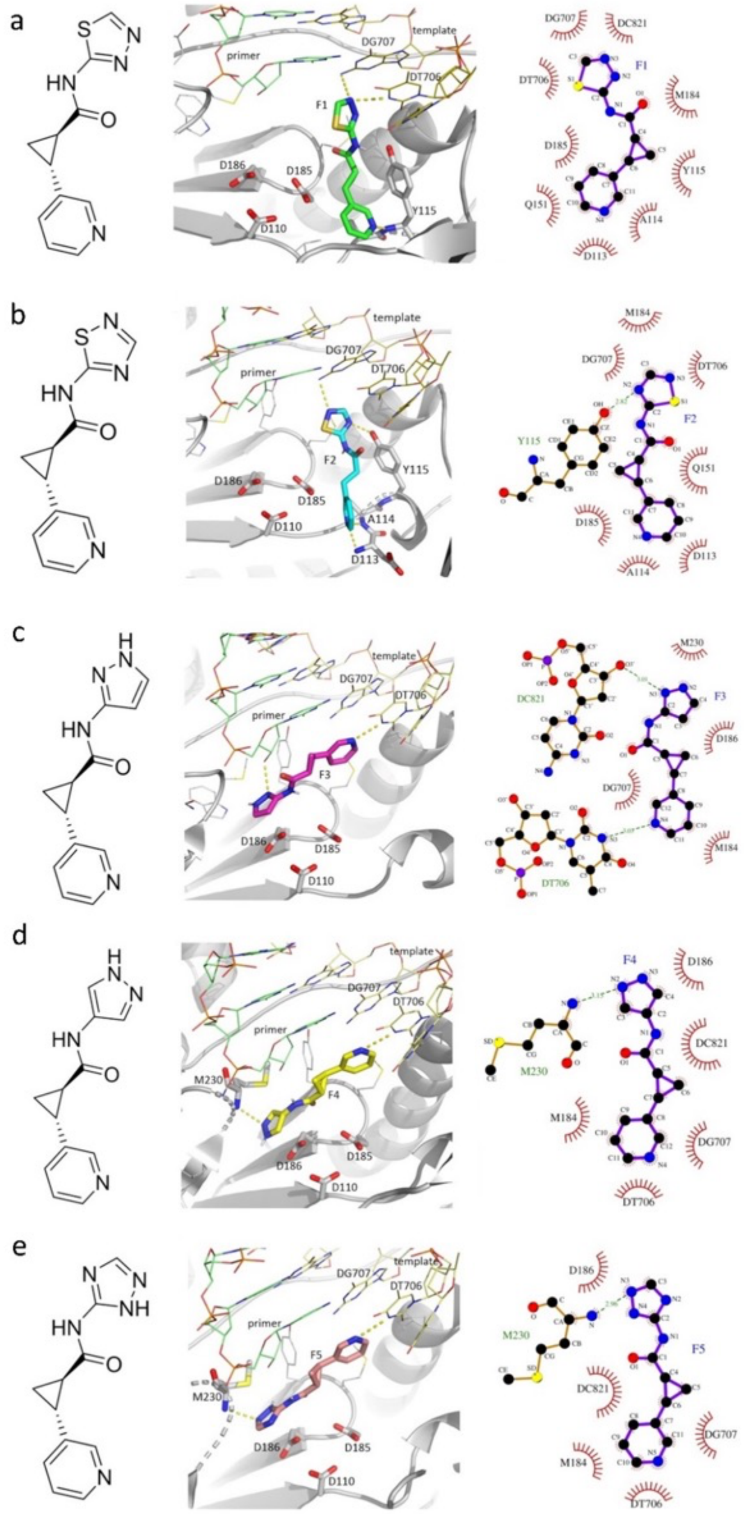

Supplementary Fig. 3. Docking mode and detailed interactions of designed fragments F01-F05 with HIV-1 RT/dsDNA (panels **a - e**). Conformation having best free energy of binding is shown in each case. Hydrogen bond (H-bond) interactions are shown with yellow dashed lines; binding poses were prepared in PyMol,[1] and detailed interaction diagrams were obtained with LigPlot+ v.2.2.[2]

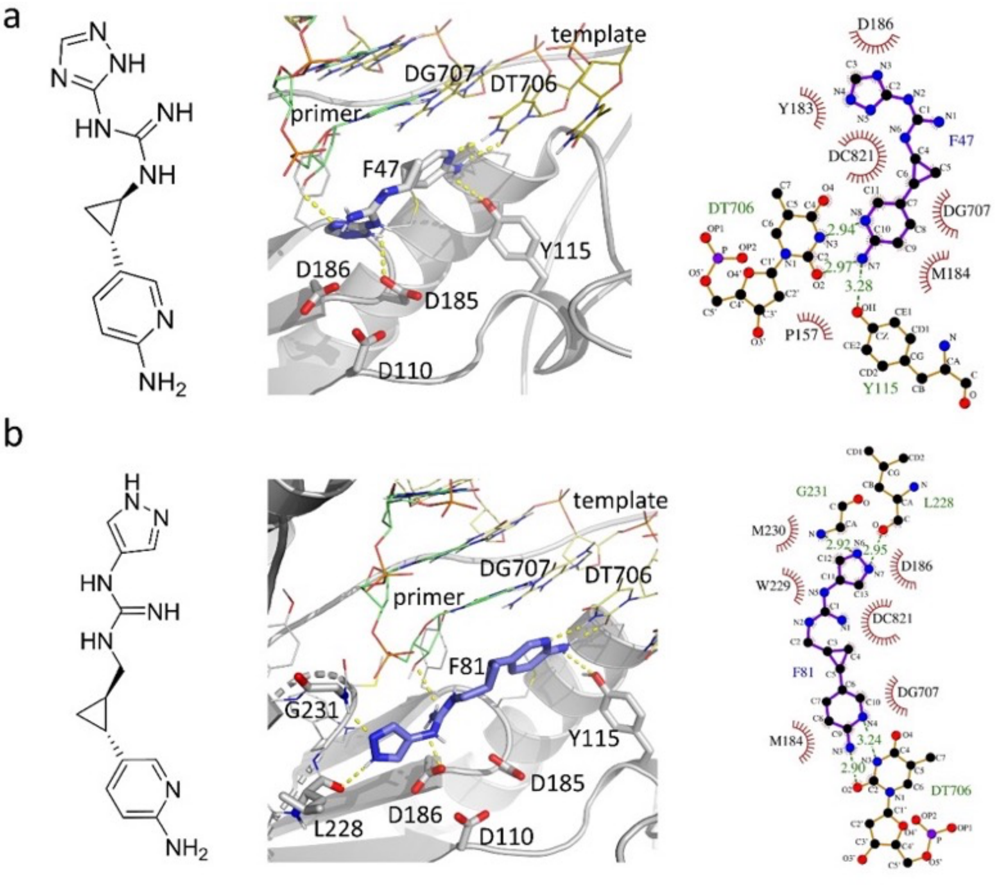

Supplementary Fig. 4. Interactions of (a) F47 and (b) F81 with HIV-1 RT/dsDNA. Conformation having best free energy of binding is shown in each case. H-bond interactions are depicted in yellow dashed lines; docking poses were generated in PyMol,[1] and detailed interaction diagrams were obtained with LigPlot+ v.2.2.[2]

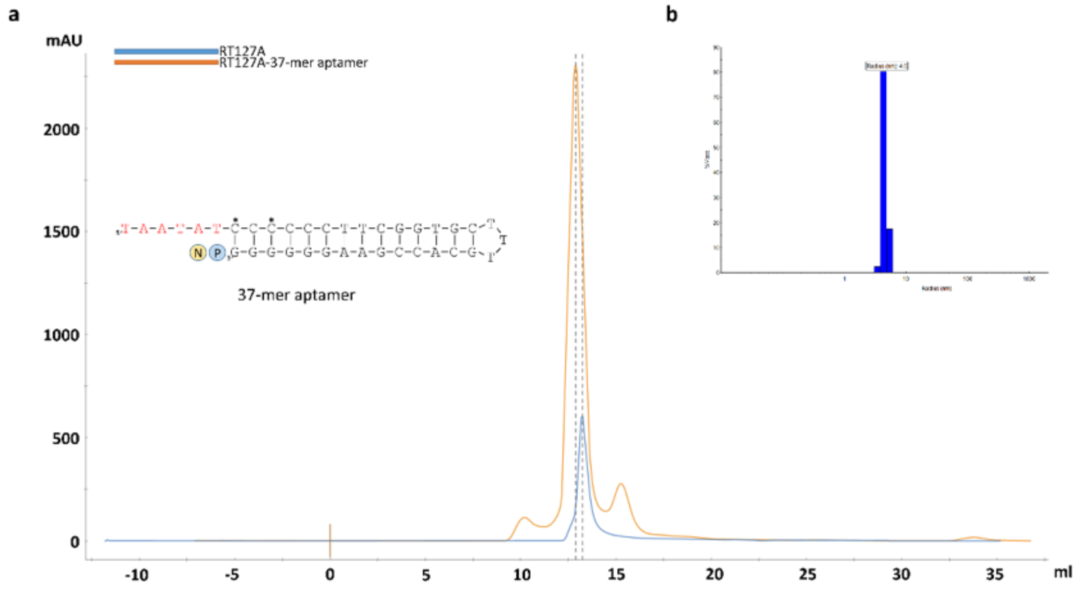

Supplementary Fig. 5. Size-exclusion chromatography of HIV-1 RT/37-mer hairpin-DNA aptamer complex. a sequence of the 37-mer hairpin-DNA aptamer. Template overhang is in red, P-site and N-site are shown under blue and yellow circles respectively. Eluted peak of the RT-aptamer complex (orange) is compared with that of the RT only peak (blue) to highlight the shift in the profile; mid points of the peaks are labelled with vertical dashed lines. **b** dynamic light scattering (DLS) profile of the RT-aptamer complex showing particle size and sample homogeneity.

Supplementary Table 4. Single particle cryo-EM data and structure analysis statistics.

Structure	RT/Aptamer DNA/**166**	RT/Aptamer/**F04**
PDB ID/EMBD ID	7OZW/EMD-13139	7P15/EMD-13156
Data collection		
Grid type	Quantifoil R1.2/1.3	Quantifoil R1.2/1.3
Number of grids	1	1
Microscope/detector	Glacios/Falcon 3	Glacios/Falcon 3
Voltage (kV)	200	200
Magnification	150,000 x	150,000 x
Recording mode	Counting	Counting
Dose (e^-/Å^2/frame)	1.25	1.25
Total dose (e/Å^2)	50	50
Number of frames/movies	40	40
Total exposure time (sec)	55	55
Pixel size (Å)	0.97	0.97
Defocus range (Å)	-8000 to - 18000	-8000 to - 18000
	Data processing	
Number of micrographs used	660	767
Number of particles picked	856,171	733,223
Particles used for final map	146,670	157,094
Fourier Completeness	0.911	0.89
Map resolution (FSC 0.143; Å)	3.38	3.58
Map sharpening B factor (Å^2)	140.2	148.9
Model fitting		
Experimental map/model correlation	0.70	0.72
Experimental map/ligand correlation	0.43	0.53
Total number of atoms	8,407	8,639
Number of residues/Average B factor (Å^2)		
Protein	961/35.08	969/55.60
Nucleic acid	34/82.97	34/94.53
Ligand	1/65.50	1/63.73
Clash score	7.07	8.4
Ramachandran plot; favored/outlier (%)	96.34/0.0	96.05/0.0
Rotamer outlier (%)	0.0	0.12
RMSD bond length (Å)/bond angle (°)	0.004/0.74	0.004/0.62
MolProbity score	1.63	1.79

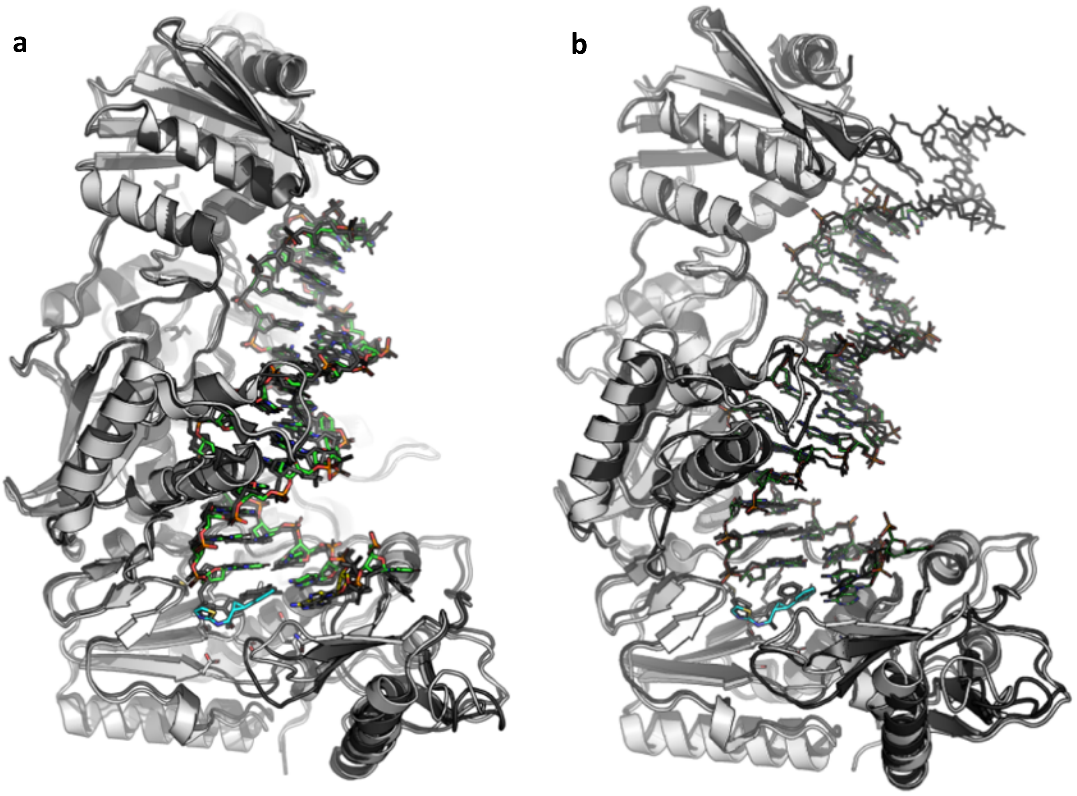

Supplementary Fig. 6. Impacts of crystallography and cryo-EM experimental conditions on the complexes. a. Superposition of cryo-EM structure of RT/37-aptamer DNA/**166** complex (light gray RT, green DNA, and cyan **166**) and crystal structure of RT/38-aptamer DNA (PDB ID. 5D3G, dark gray) revealed that the structural features including the track of DNA aptamer, subdomain arrangements, and DNA-protein interactions are conserved between two structures. The fragment **166** is bound to P-pocket in P-1 complex cryo-EM structure, and the pocket is occupied by the 3'-end nucleotide of aptamer DNA in the crystal structure. A total of 866 Cα atoms superimposed with rmsd of 0.95 Å. **b.** The cryo-EM structure of RT/37-aptamer DNA/**166** complex (light gray RT, green DNA, and cyan **166**) and crystal structure of cross-linked RT/DNA/**166** complex (dark gray) superimpose well with rmsd of 1.14 Å for 931 aligned Cα atoms.

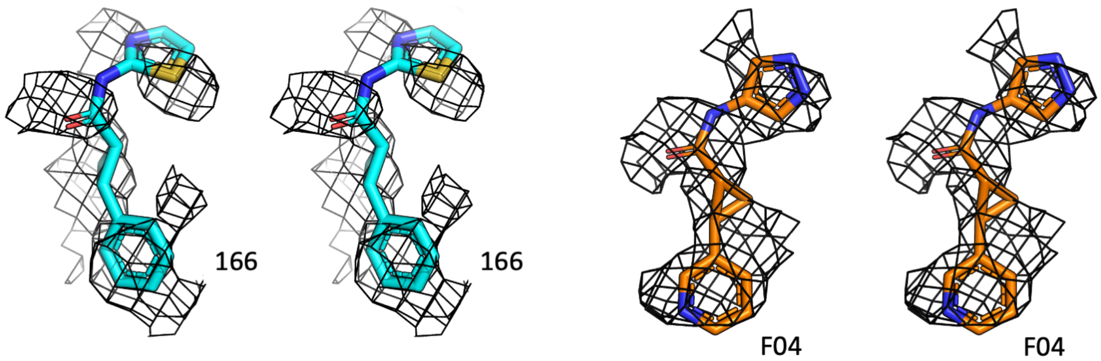

166 166 F04 F04

Supplementary Fig. 7. Stereo views of cryo-EM density for the compounds **166** (left) and **F04** (right) in respective structures in complexes with HIV-1 RT. The maps are contoured at 1.2σ and 1.5σ, respectively.

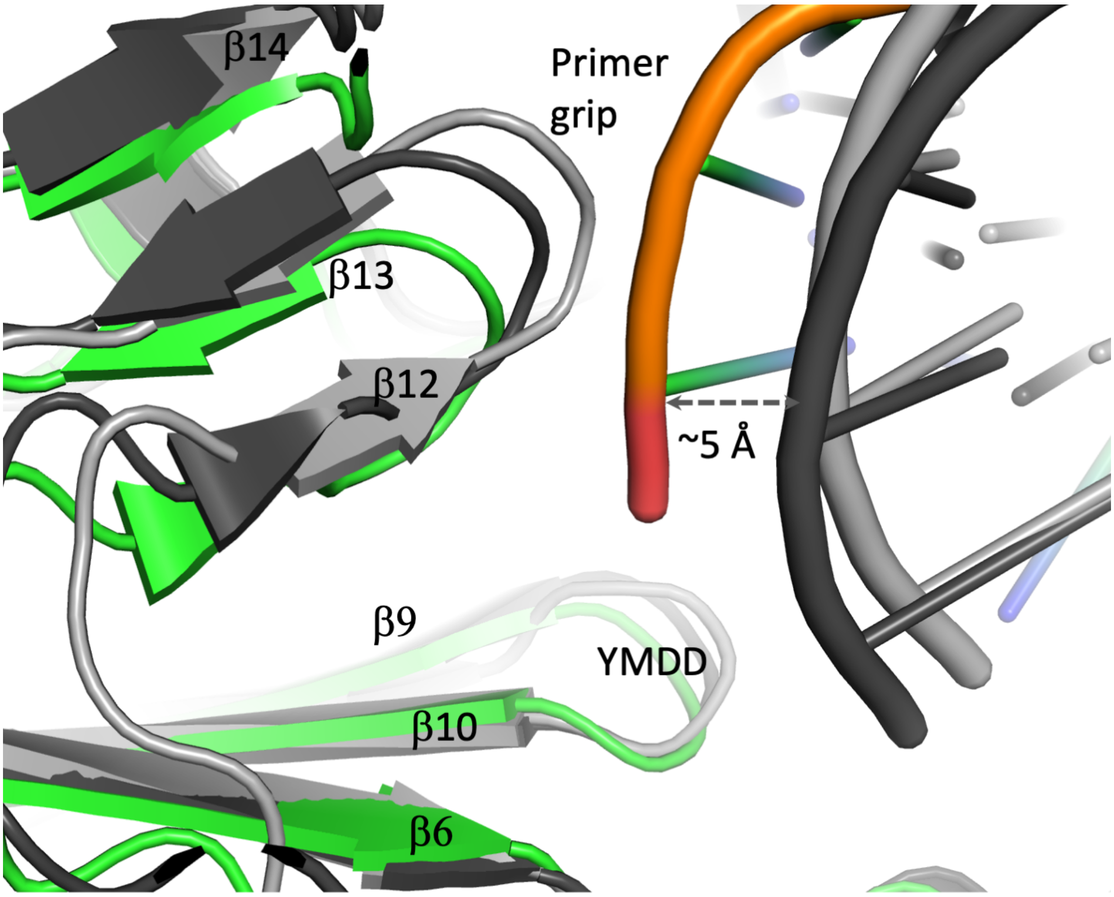

Supplementary Figure 8. Superstitions of RT/dsRNA cryo-EM structure (PDB Id. 7KJV; light gray) and crystal structure (PDB Id. 6HAK; dark gray) on apo P-1 complex (green) shows a different track for dsRNA compared to dsDNA (orange); the 3'-end of DNA primer and the corresponding RNA primer P-1 nucleotides are about 5 Å apart.

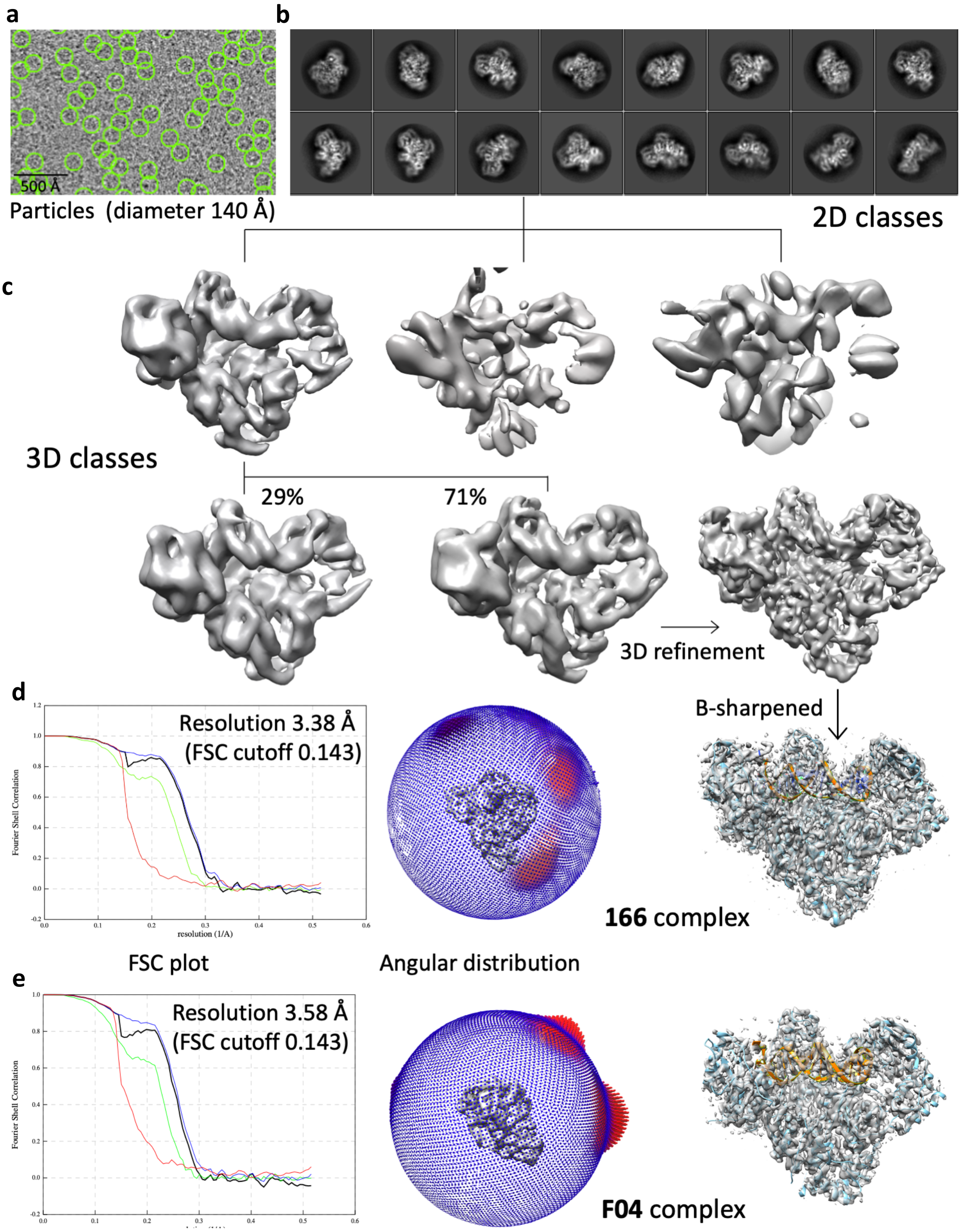

Supplementary Fig. 9. Cryo-EM data processing. a. Picked particles on a typical micrograph of RT/aptamer-DNA/166 complex. **b**. Selected 2D classes. **c.** 3-D classes of RT/DNA/166 complex. The first 3D classification found one class of intact particles and the reclassification of this class grouped 71% of particles to a homogenous class; the remaining 29% particles were less homogenous and did not represent a distinct structural state of the complex. FSC resolution, angular distribution of particles in the final set, and B-sharpened map covering the model for RT/aptamer-DNA/166 complex structure (row **d**) and RT/aptamer-DNA/F04 complex structure (row **e**).

a

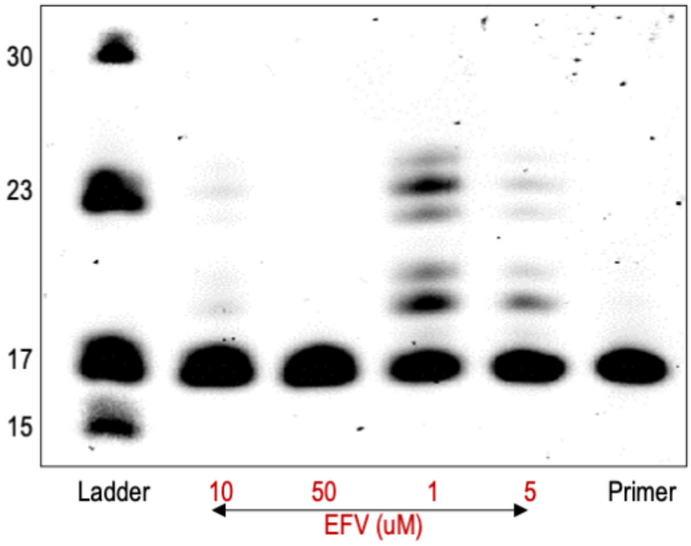

b

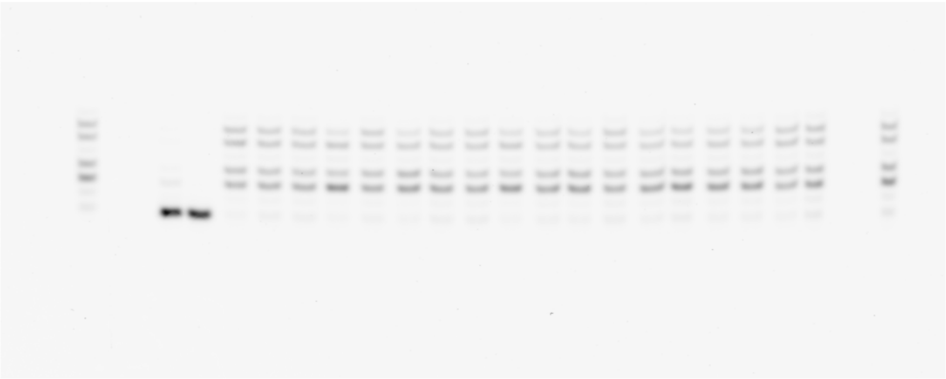

Supplementary Fig. 10. RT inhibition assay supporting figures. a A ladder with markers at 15, 17, 23, and 30 nucleotides was included in assay showing inhibition by efavirenz (EFV) for calibration of the gel in Fig. 5g. **b** The original gel of the one shown in Fig 5g.

Supplementary Methods

Fragment design and docking study

Creation of a well-formed P-pocket and the binding of the fragments **048** and **166** to the pocket intuited the need for further analysis of the pocket characteristics. We attempted virtually designing a set of compounds with the chemical backbone of **166**, such that the analogs can form H-bond with template thymine base. Therefore, the phenyl ring was replaced by a pyridine moiety. In addition, an amino or hydroxyl group was introduced at the 5'-position of the pyridine ring so that two H-bonds could be potentially formed like base-pairing. At the same time, we maintained the amide bond of **166**, and made variations of the thiazole moiety with different five-membered heteroaromatics. Eighteen (**F01-F18**) analogs of **166** were modeled by accommodating the above discussed variations (Supplementary Table 1).

In order to investigate the favorability of binding of the designed fragments to P-pocket, we conducted molecular docking study; initially rigid docking followed by flexible docking using Autodock Vina (ver. 1.1.1).[3] For flexible docking, we defined the catalytic residues D110, D185 and D186 of HIV-1 RT as flexible residues. Analysis of docking results revealed that ten fragments (**F03-F08, F10-F13**) have the potential for forming H-bonds with template thymine overhang. In addition, the newly introduced heteroaromatics can engage in additional H-bond formation with surrounding residues and DNA primer. Specifically, fragments **F03, F8, F10-11** formed one H-bond with 3'-hydroxyl group of the primer end nucleotide DC821, while **F04-F06** formed one H-bond with backbone NH of primer grip residue M230. Moreover, **F07, F12-13** formed H-bonds with both M230 and the primer 3'-end nucleotide. However, all above-mentioned fragments did not acquire H-bond interactions with catalytic residues D110, D185 and/or D186. Based on these observations and taking into account the synthesis feasibility, we proceeded to synthesize fragments **F01-F05** whose docking outcomes are shown (Supplementary Figure 1, Supplementary Table 2).

The guanidinium group as present in the side-chain of arginine interacts with carboxylate groups via salt bridges, which can be found in many crystal structures of enzyme complexes with oxoanionic substrates and simple guanidinium salts.[4] To explore the possibility of our compounds forming such interactions with the catalytic residues, we decided to substitute the amido linkage for an amidine or guanidine linker and make small variation on the linker length to increase flexibility, resulting in the design of the fragments **F19-F84** (Supplementary Table 1).

Molecular docking study demonstrated that fragments **F19-F24** with an amidine linker can form H-bonds with the template thymine and with the residue D185, whereas **F26-F30** form H-bonds with thymine only. Fragments (**F43, F45-46** and **F52**) with a guanine linker can only form H-bonds with template thymine, whereas **F47** and **F48** can also interact with D185. Interestingly, although fragments (F62-F64) with longer guanine linker only form H-bonds with thymine, their counterparts (F60, F66, F79-F84) can also form one additional interaction with the residue D186. For these fragments, the newly introduced heteroaromatics (Supplementary Table 1) can also form H-bond with the primer grip/primer 3'-nucleotide. Both **F81** and **F82** can form one H-bond with 3'-hydroxyl end of the primer strand and backbone of residue L228, respectively, and the former can form an additional H-bond with G231 while maintaining interaction with the template thymine.

Considering the binding affinity, stability and structural variation of designed fragments, and ease of synthesis we selected two fragments (**F47** and **F81**) for synthesis. The detailed docking results and drug-like properties of hits (**048** and **166**) and selected fragments are shown in Supplementary Table 2. Docking score of selected fragments were improved when compared with that of two fragment screen hits. In addition, the docked modes of **F47** and **F81** suggested more interacting surface areas than that of two hits (Supplementary Fig. 2). Furthermore, selected fragments satisfy Lipinski's Rule of Five.[5]

Synthesis of fragments

In view of their convenient synthesis, we firstly prepared **F01-05** for structural study to examine if the pyridyl ring can form H-bond with template thymine. The preparation of these compounds is depicted in Supplementary Fig. 10. The *trans*-cyclopropane-containing compounds were all synthesized as racemic mixtures.

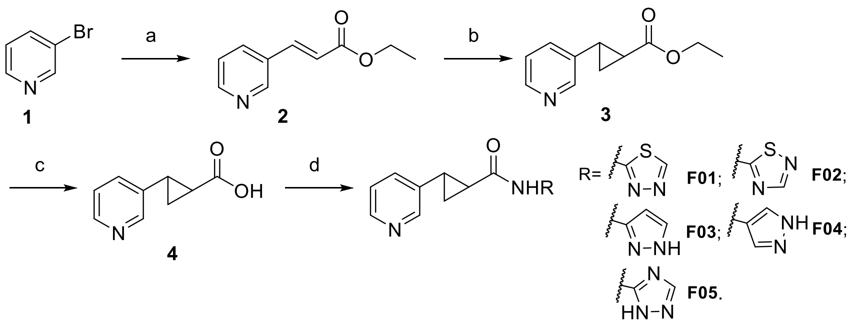

Supplementary Fig. 11. *Reagents and conditions*: (a) Ethyl acrylate, $Pd(OAc)_2$, K_2CO_3, PPh_3, DMF, 100 °C, 20 h, 75 %; (b) Me_3SOI, NaH, DMSO, r.t., 1 h, 26 %; (c) NaOH, MeOH, H_2O, 60 °C, 12 h, 49 %; (d) RNH_2, EDCI, DIEA, HOAt, DMF, -20 °C to r.t., overnight, 20%-52 %.

Although the synthetic route for cyclopropanation is shorter in Supplementary Fig. 11, the yield is low (20-30 %). Thus, to prepare two selected fragments, we proposed another synthetic route modified from a reported sequence of reactions,[6] from which the yield reached 82 %. The synthesis of fragment **F47** is illustrated in Supplementary Fig. 12. In order to remove protecting groups in the last step, strongly acidic condition was attempted firstly, but no desired compound was obtained. The high-resolution mass spectra (HRMS) of main product indicated that PMB groups were removed successfully, but the guanidine moiety unexpectedly formed a six-membered ring with triazole ring (Supplementary Figure 13). Alternatively, reductive hydrogeneration employing Pd/C

catalyst was attempted to remove Cbz group, but no new product was detected. Then, we moved on to the synthesis of **F81**. When the protecting groups in compound **16** were attempted to remove under strongly acidic condition, HRMS of the main product again showed that PMB groups were removed, whereas the guanidine moiety also formed a six-membered ring with pyrazole ring (Supplementary Figure 14).

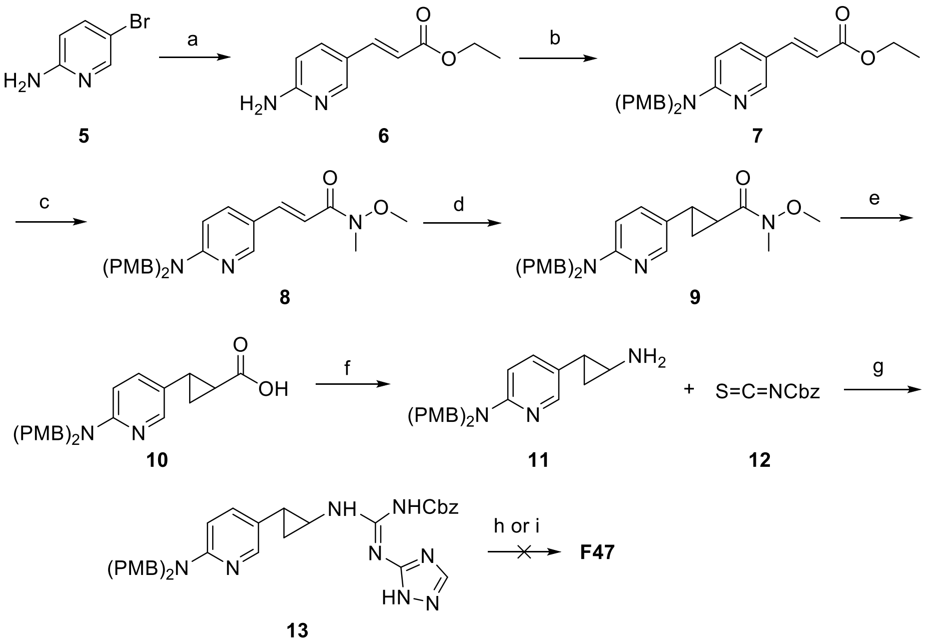

Supplementary Fig. 12. Synthesis of F47. *Reagents and conditions*: (a) Ethyl acrylate, $Pd(OAc)_2$, DIEA, $P(o\text{-}tol)_3$, DMF, 100 °C, 20 h, 74 %; (b) PMB-Cl, NaH, DMF, 0 °C, 1 h, 71 %; (c) (i) 2M NaOH in EtOH/H_2O, r.t., 24 h; (ii) N,O-dimethylhydroxylamine hydrochloride, EDCI, DMAP, DCM, r.t., 2 h, 40 % over two steps; (d) Me_3SOI, NaH, DMSO, 0 °C to r.t., 4 h, 82 %; (e) KOH, EtOH/H_2O, r.t., 24 h, 62 %; (f) (i) DPPA, TEA, benzene, 80 °C, 1 h; (ii) H_2O, 80 °C, 30 min, 43 % over two steps; (g) (i) DCM, 0 °C to r.t., 4 h; (ii) 1,2,4-triazol-5-amine, EDCI, DIEA, DCM, 0 °C, 1h, then r.t.,10 h, 32 % over two steps; (h) TFA, DCM, r.t., 5 h; (i) 10 % Pd/C, MeOH, r.t., 24 h.

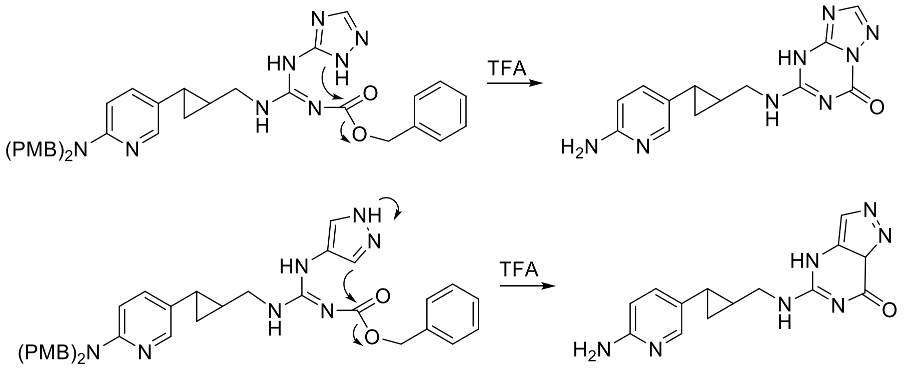

Supplementary Figure 13. Proposed formation of by-product indicated by HRMS.

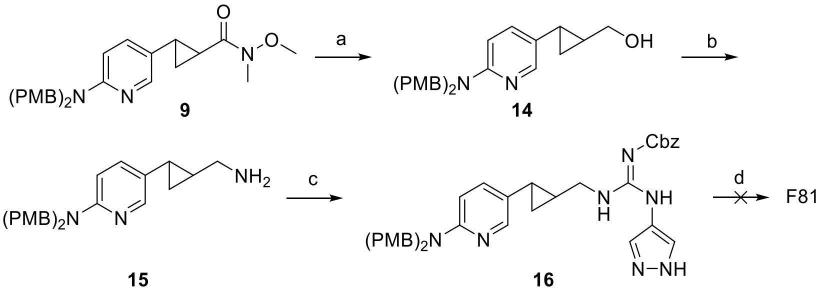

Supplementary Fig. 14. Synthesis of F81. *Reagents and conditions*: (a) $LiAlH_4$, THF, 0 °C, 2 h, 72 %; (b) (i) MsCl, TEA, DCM, 0 °C, 2 h; (ii) NaN_3, DMF, 60 °C, 6 h; (iii) PPh_3, THF, H_2O, r.t., 12 h, 25 % over three steps; (c) (i) DCM, 0 °C to r.t., 4 h; (ii) pyrazol-4-amine, EDCI, DIEA, DCM, 0 °C, 1 h, then r.t.,10 h, 63 % over two steps; (d) TFA, DCM, r.t., 5 h.

Supplementary Figure 15. NMR spectra and HRMS of fragments F01-05

Spectra of **F01**

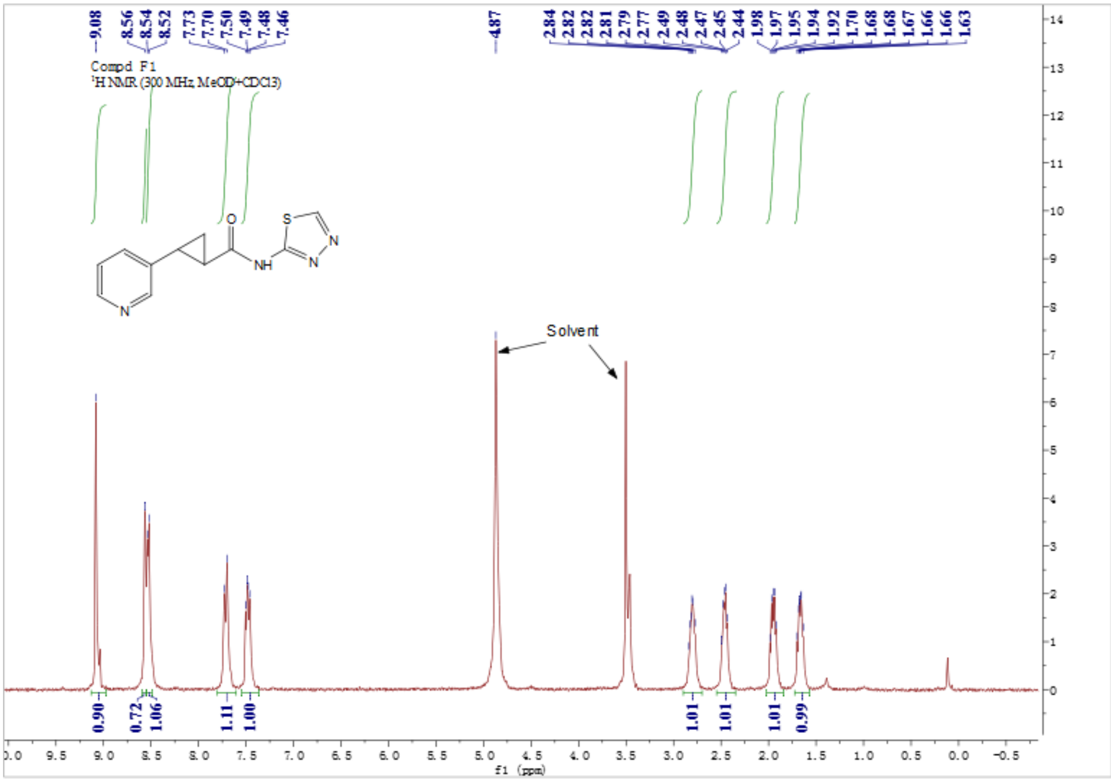

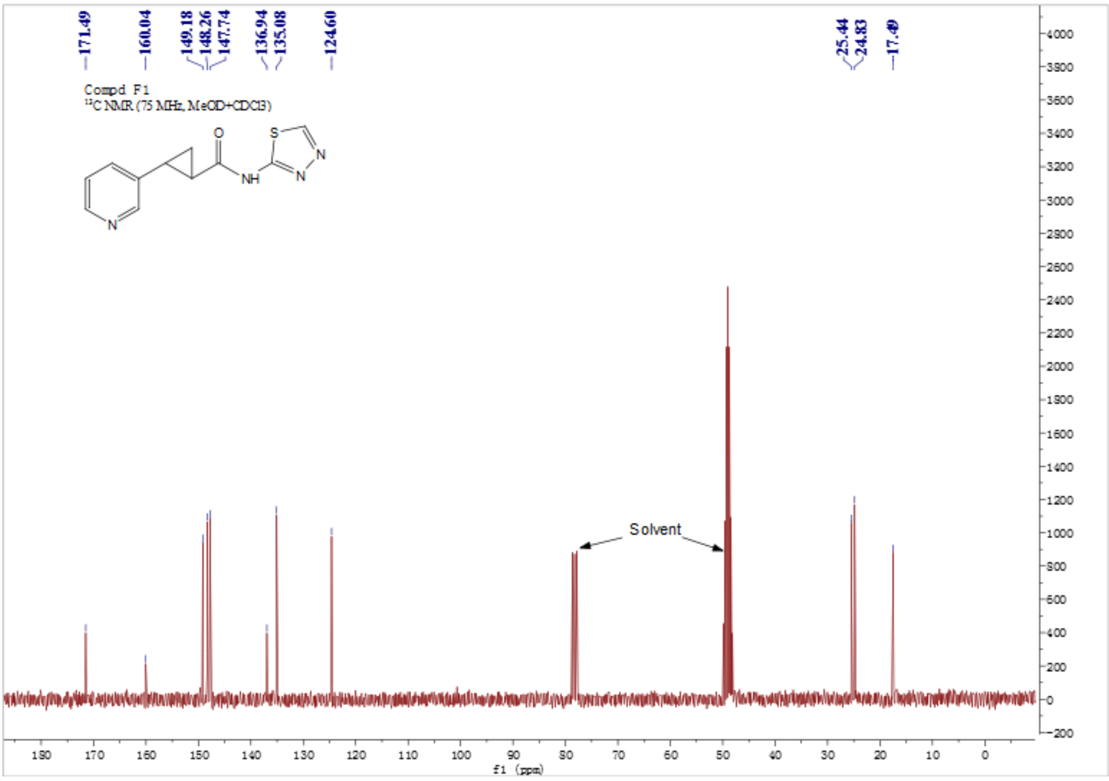

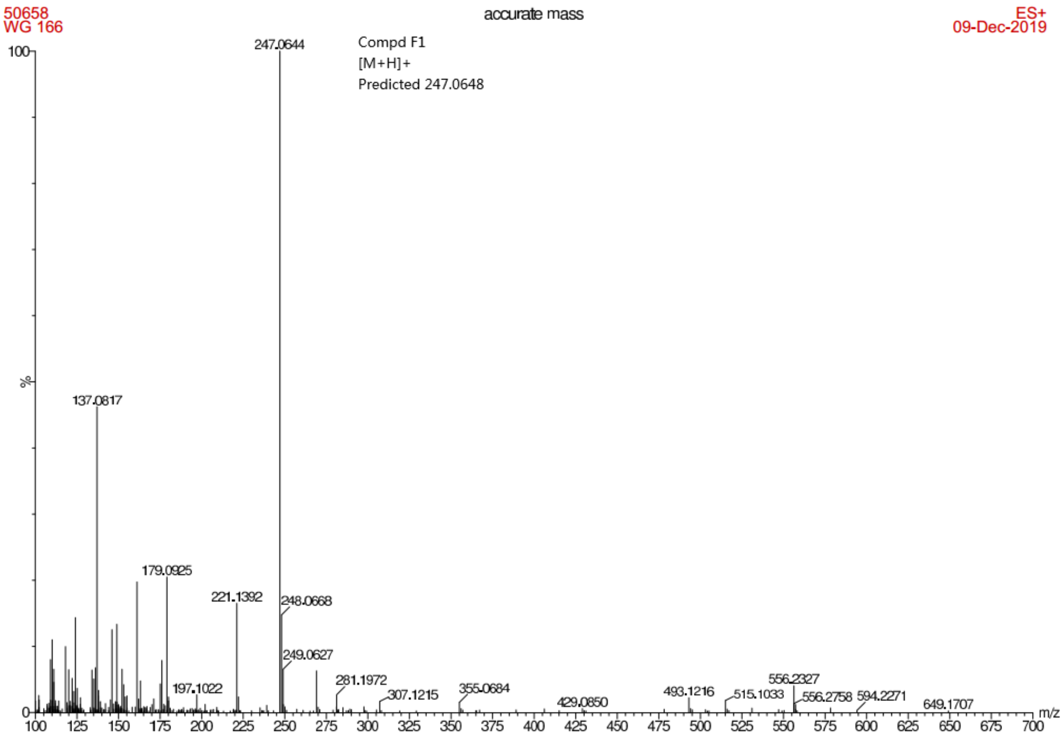

Spectra of **F02**

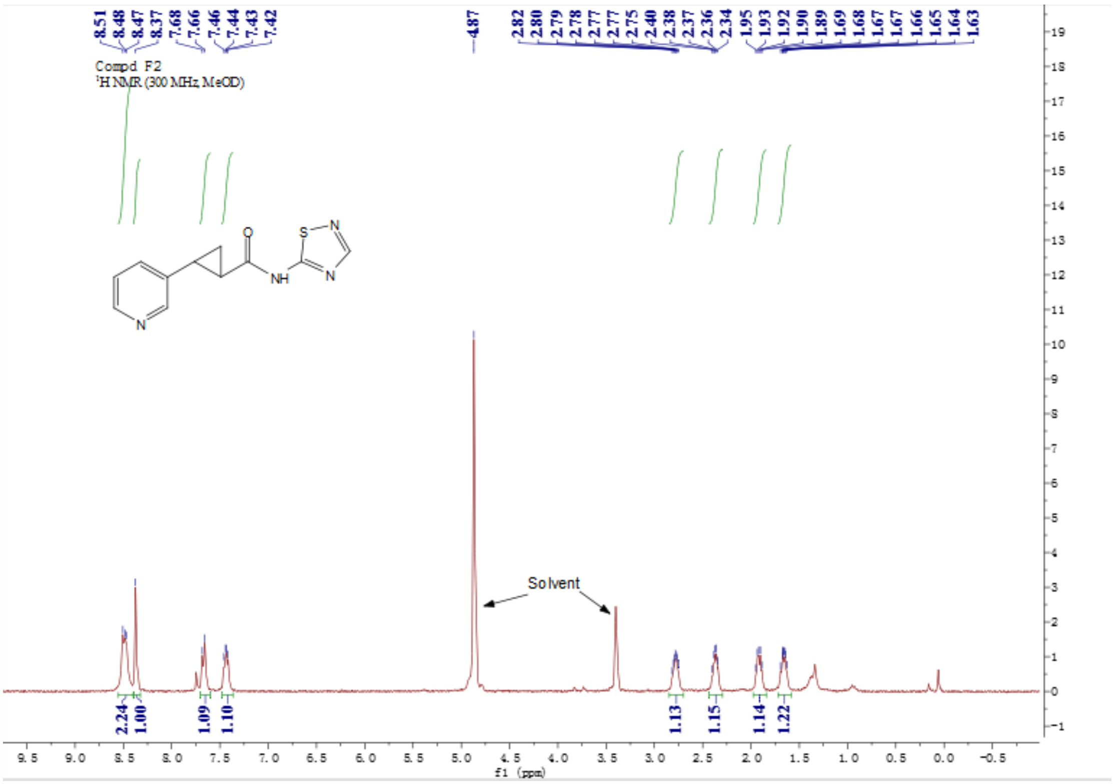

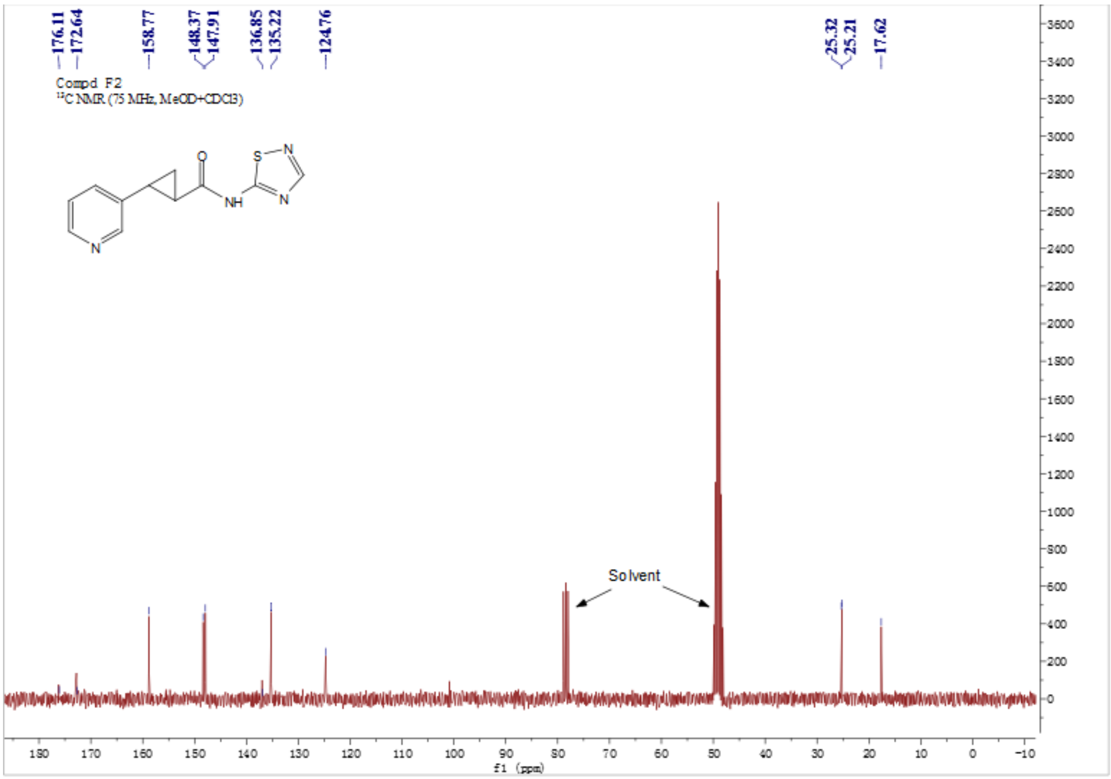
176.11
172.64
158.77
148.37
147.91
136.85
135.22
124.76
25.32
25.21
17.62
Compd F2
13C NMR (75 MHz, MeOD+CDCl3)
Solvent
f1 (ppm)

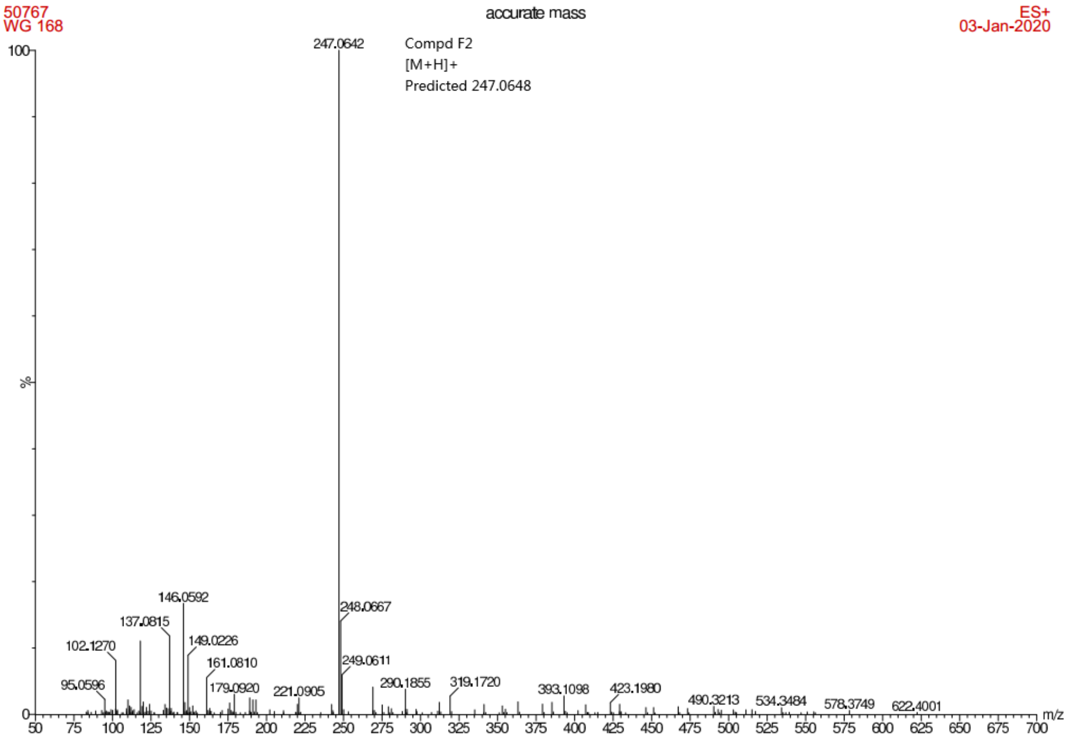
50767
WG 168
accurate mass
ES+
03-Jan-2020
247.0642
Compd F2
[M+H]+
Predicted 247.0648
146.0592
137.0815
102.1270
149.0226
161.0810
95.0596
179.0920
221.0905
248.0667
249.0611
290.1855
319.1720
393.1098
423.1980
490.3213
534.3484
578.3749
622.4001
m/z
%

Spectra of **F03**

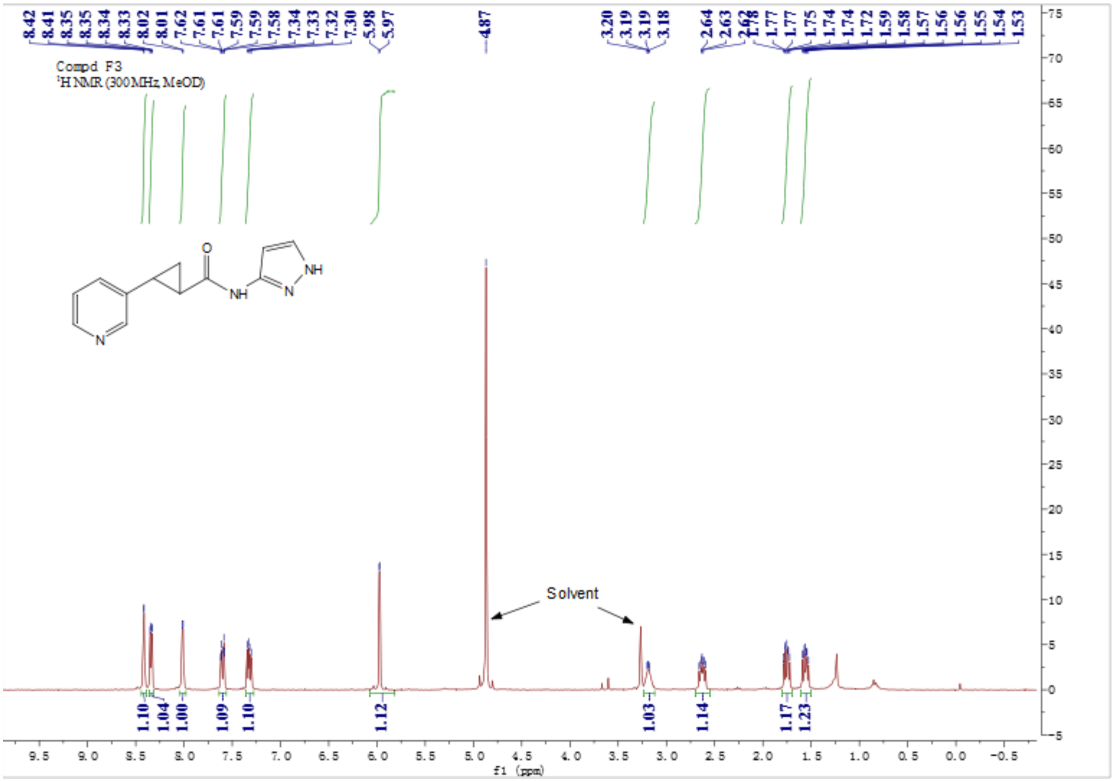

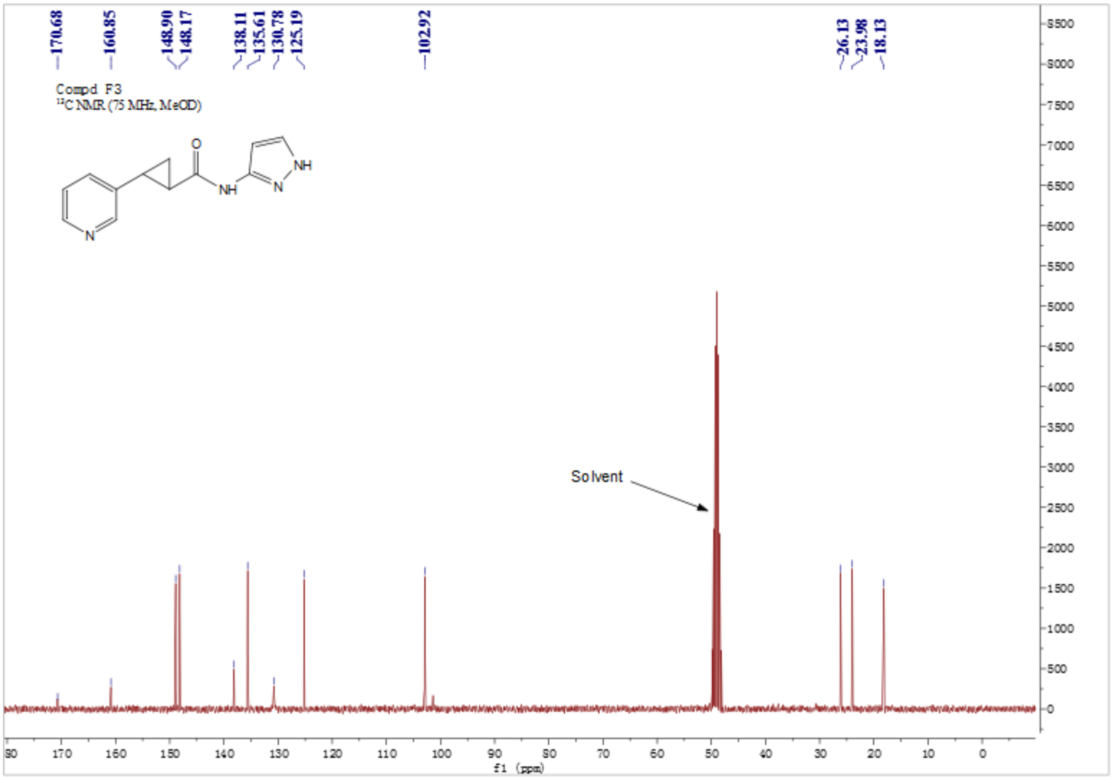

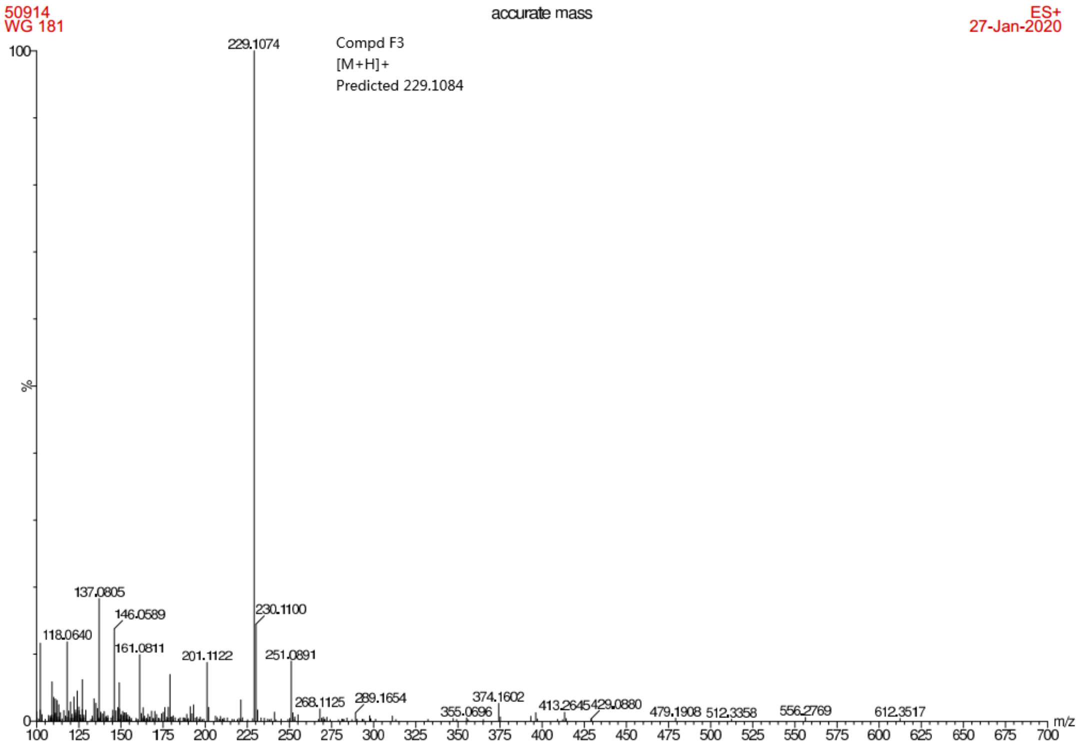

Spectra of **F04**

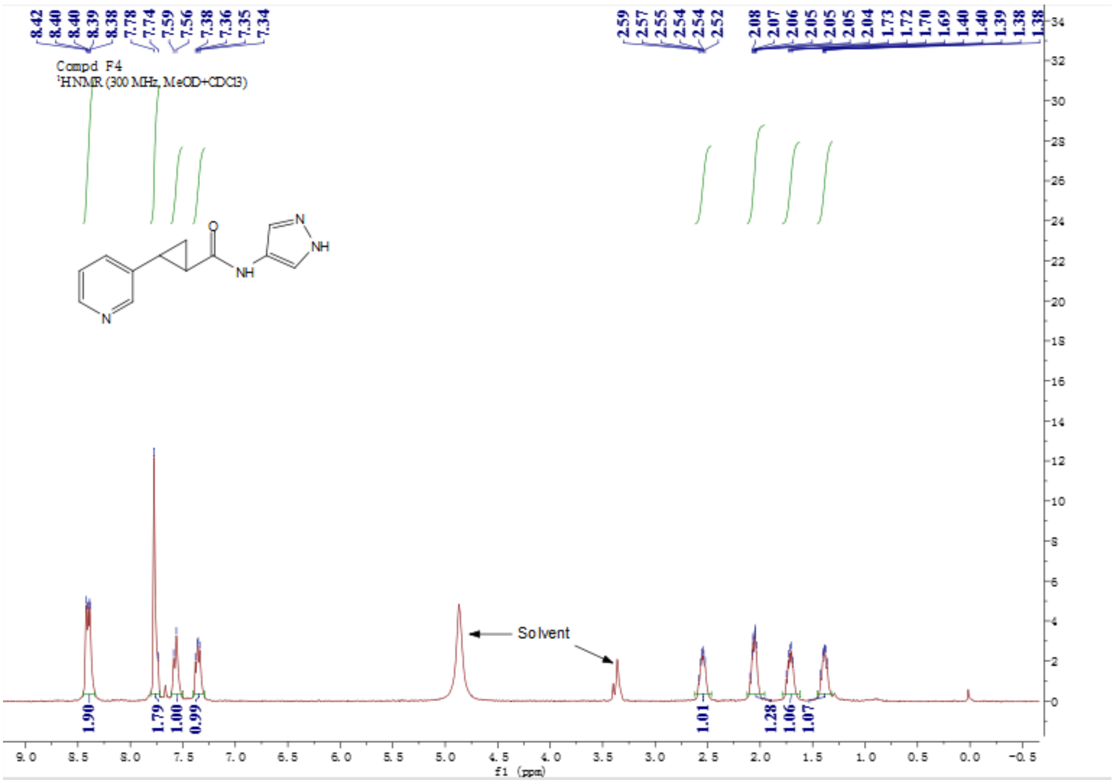

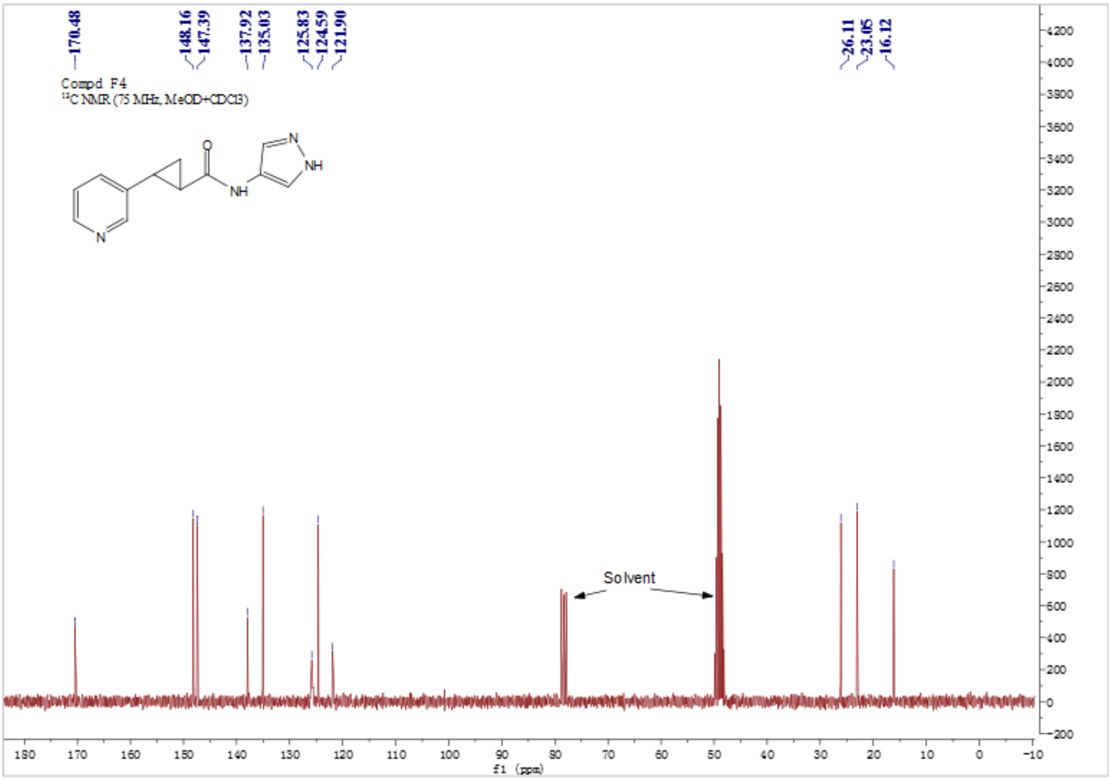
170.48
148.16
147.39
137.92
135.03
125.83
124.59
121.90
26.11
23.05
16.12
Compd F4
13C NMR (75 MHz, MeOD+CDCl3)
Solvent
f1 (ppm)

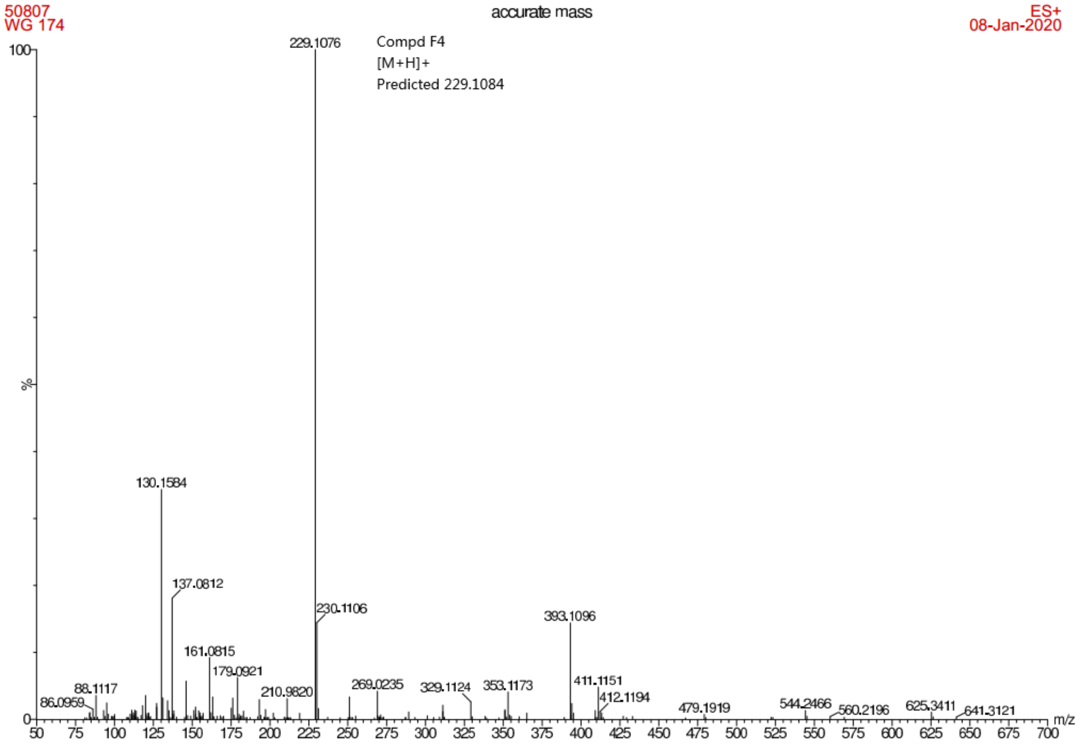
50807
WG 174
accurate mass
ES+
08-Jan-2020
Compd F4
[M+H]+
Predicted 229.1084
229.1076
130.1584
137.0812
230.1106
393.1096
161.0815
179.0921
210.9820
269.0235
329.1124
353.1173
411.1151
412.1194
86.0959
88.1117
479.1919
544.2466
560.2196
625.3411
641.3121
m/z
%

Spectra of **F05**

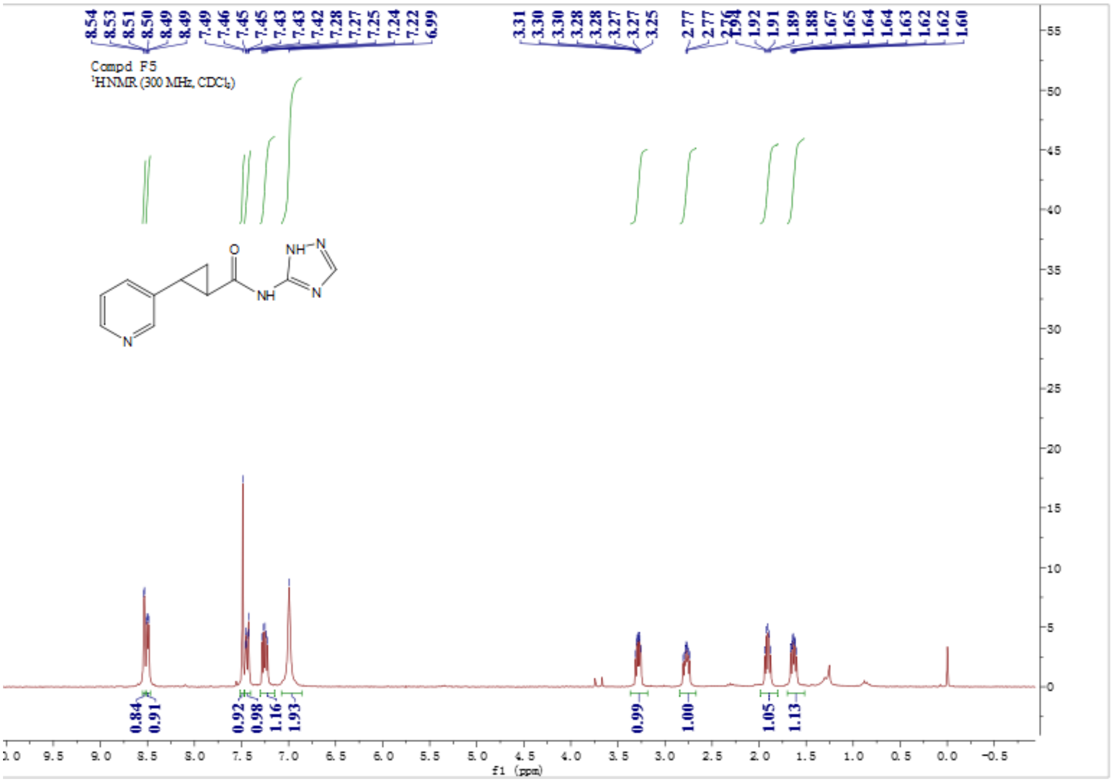

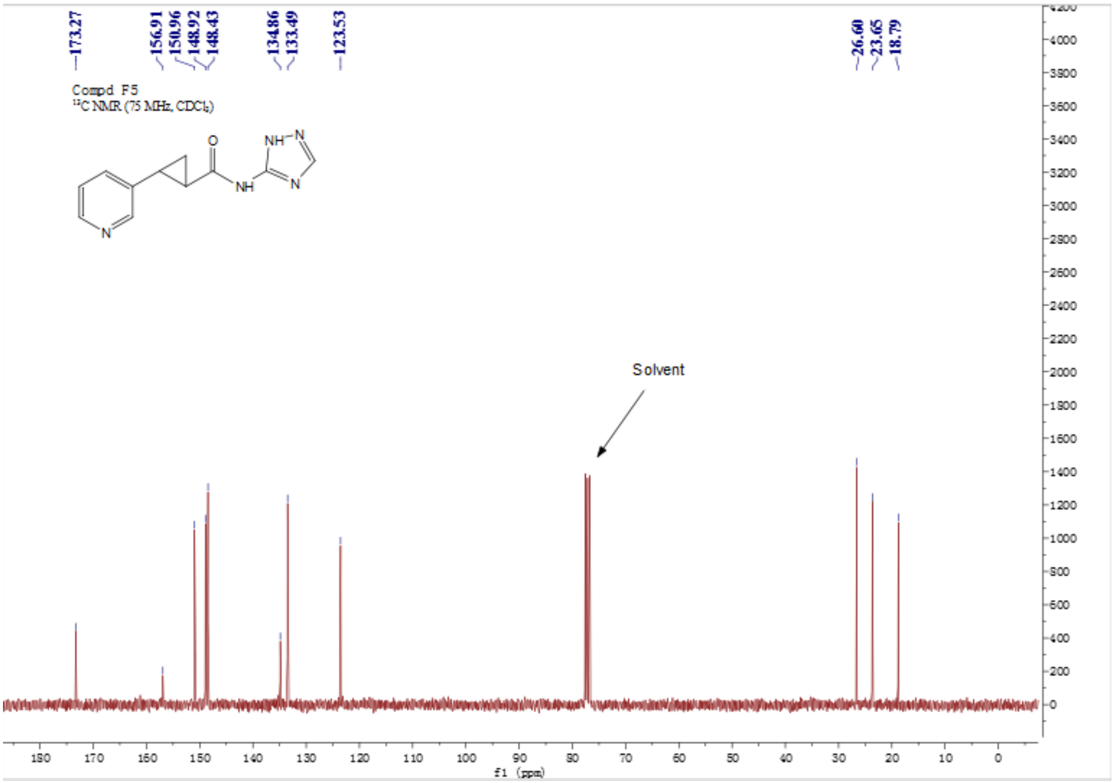

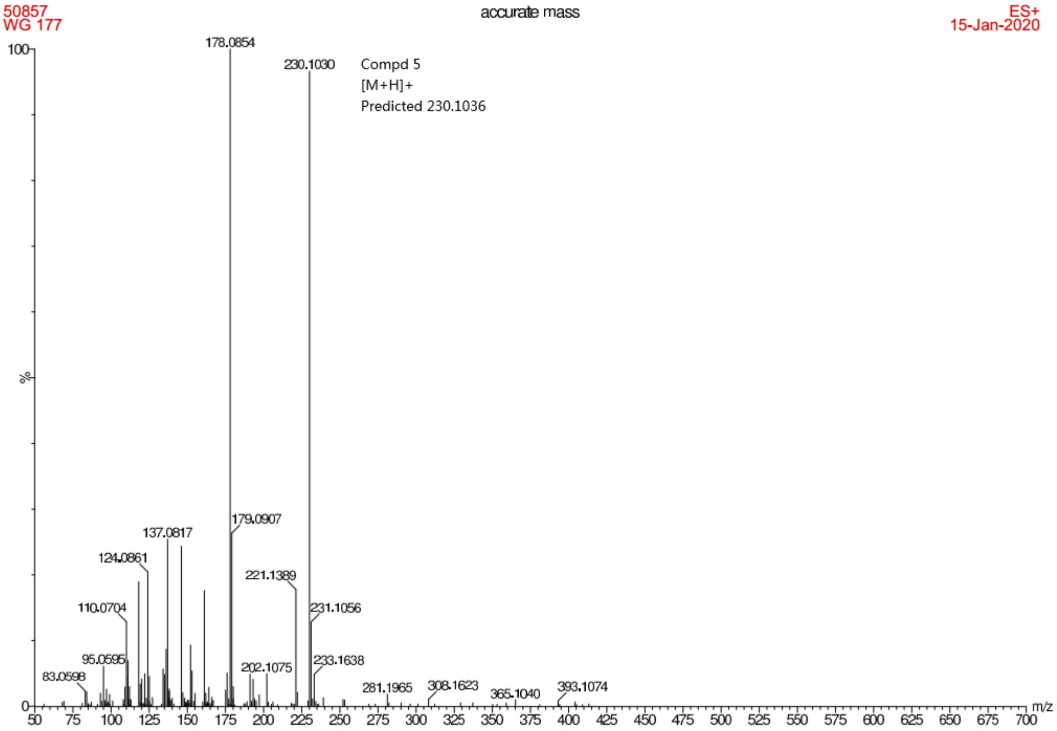

50857
WG 177
accurate mass
ES+
15-Jan-2020
178.0854
230.1030
Compd 5
[M+H]+
Predicted 230.1036
179.0907
137.0817
124.0861
221.1389
110.0704
231.1056
95.0595
233.1638
83.0598
202.1075
281.1965
308.1623
365.1040
393.1074
%
m/z

References

1. The PyMOL Molecular Graphics System, Version 1.8 Schrödinger, LLC.
2. Laskowski, R. A.; Swindells, M. B. LigPlot+: multiple ligand-protein interaction diagrams for drug discovery. *J. Chem. Inf. Model* **2011,** 51, 2778-2786.
3. Trott, O.; Olson, A. J. AutoDock Vina: improving the speed and accuracy of docking with a new scoring function, efficient optimization and multithreading. *J. Comput. Chem.* **2010,** 31, 455-461.
4. F. P. Schmidtchen; Berger, M. Artificial Organic Host Molecules for Anions. *Chem. Rev.* **1997,** 97, 1609-1646.
5. Lipinski, C. A. Drug-like properties and the causes of poor solubility and poor permeability. *Journal of Pharmacological and Toxicological Methods* **2000,** 44, 235-249.
6. Giannetti, A. M.; Zheng, X.; Skelton, N. J. *et al.* Fragment-based identification of amides derived from trans-2-(pyridin-3-yl)cyclopropanecarboxylic acid as potent inhibitors of human nicotinamide phosphoribosyltransferase (NAMPT). *J Med Chem* **2014,** 57, 770-92.
