# Supplementary material for: Sliding of HIV-1 reverse transcriptase over DNA creates a transient P pocket – targeting P-pocket by fragment screening
Source: Nat Commun. 2021 Dec 8;12:7127. doi: 10.1038/s41467-021-27409-y (PMC8654897; doi:10.1038/s41467-021-27409-y)
Supplement: Supplementary file 3 — Description of Additional Supplementary Files [file 41467_2021_27409_MOESM3_ESM.pdf]

Description of Additional Supplementary Files

File Name: Supplementary Movie 1
Description: Sliding of RT over a dsDNA substrate. The structures of N, P, and P-1 complexes of I63C RT/DNA were morphed to show sliding of RT over a dsDNA. The movie shows the transition starting from the N complex (light gray) to P-1 complex (salmon) via P complex; in the movie, the position of RT is fixed, and the DNA is sliding. The fingers were closed in N and P complex as the N-site (dNTP-binding pocket) is occupied in those structures.

File Name: Supplementary Movie 2
Description: A closer view at the polymerase active site. The color code and sliding are as in Movie 1.

File Name: Supplementary Movie 3
Description: Rearrangement of P-pocket to accommodate the fragment 166. Morphing between the fragment 166-bound RT (salmon) and apo RT (light gray) structures show the rearrangement of P pocket to bind the fragment.
