# Supplementary material for: Sliding of HIV-1 reverse transcriptase over DNA creates a transient P pocket – targeting P-pocket by fragment screening
Source: Nat Commun. 2021 Dec 8;12:7127. doi: 10.1038/s41467-021-27409-y (PMC8654897; doi:10.1038/s41467-021-27409-y)
Supplement: Supplementary file 7 — Reporting Summary [file 41467_2021_27409_MOESM7_ESM.pdf]

nature portfolio

Corresponding author(s): Kalyan Das

Last updated by author(s): Nov 7, 2021

Reporting Summary

Nature Portfolio wishes to improve the reproducibility of the work that we publish. This form provides structure for consistency and transparency in reporting. For further information on Nature Portfolio policies, see our Editorial Policies and the Editorial Policy Checklist.

Statistics

For all statistical analyses, confirm that the following items are present in the figure legend, table legend, main text, or Methods section.

n/a	Confirmed	
☒	☐	The exact sample size (n) for each experimental group/condition, given as a discrete number and unit of measurement
☒	☐	A statement on whether measurements were taken from distinct samples or whether the same sample was measured repeatedly
☒	☐	The statistical test(s) used AND whether they are one- or two-sided<br>*Only common tests should be described solely by name; describe more complex techniques in the Methods section.*
☒	☐	A description of all covariates tested
☒	☐	A description of any assumptions or corrections, such as tests of normality and adjustment for multiple comparisons
☒	☐	A full description of the statistical parameters including central tendency (e.g. means) or other basic estimates (e.g. regression coefficient) AND variation (e.g. standard deviation) or associated estimates of uncertainty (e.g. confidence intervals)
☒	☐	For null hypothesis testing, the test statistic (e.g. F, t, r) with confidence intervals, effect sizes, degrees of freedom and P value noted<br>*Give P values as exact values whenever suitable.*
☒	☐	For Bayesian analysis, information on the choice of priors and Markov chain Monte Carlo settings
☒	☐	For hierarchical and complex designs, identification of the appropriate level for tests and full reporting of outcomes
☒	☐	Estimates of effect sizes (e.g. Cohen's d, Pearson's r), indicating how they were calculated

Our web collection on statistics for biologists contains articles on many of the points above.

Software and code

Policy information about availability of computer code

Data collection	EPU software version 2.9.0 (ThermoFisher Scientific) was used for cryo-EM data collection.
Data analysis	Softwares Relion version 3.1 and Phenix version 1.19 were used for cryo-EM and crystallography data analysis, respectively.

For manuscripts utilizing custom algorithms or software that are central to the research but not yet described in published literature, software must be made available to editors and reviewers. We strongly encourage code deposition in a community repository (e.g. GitHub). See the Nature Portfolio guidelines for submitting code & software for further information.

Data

Policy information about availability of data

All manuscripts must include a data availability statement. This statement should provide the following information, where applicable:

- Accession codes, unique identifiers, or web links for publicly available datasets
- A description of any restrictions on data availability
- For clinical datasets or third party data, please ensure that the statement adheres to our policy

The coordinates and structure factors for the crystal structures of I63C RT/DNA, and its complexes with the fragments 048 and 166 are deposited in Protein Data Bank (PDB) with accession codes 7OZ2 (https://www.rcsb.org/structure/unreleased/7OZ2), 7OXQ (https://www.rcsb.org/structure/unreleased/7OXQ) and 7OZ5 (https://www.rcsb.org/structure/unreleased/7OZ5), respectively. The coordinates and cryo-EM density maps for the structures RT/DNA aptamer/166 and RT/DNA aptamer/F04 complexes are deposited with PDB accession codes/EMDB codes 7OZW/EMD-13139 (https://www.rcsb.org/structure/unreleased/7OZW) and 7P15/EMD-13156 (https://www.rcsb.org/structure/unreleased/7P15), respectively.

Field-specific reporting

Please select the one below that is the best fit for your research. If you are not sure, read the appropriate sections before making your selection.

- [x] Life sciences
- [ ] Behavioural & social sciences
- [ ] Ecological, evolutionary & environmental sciences

For a reference copy of the document with all sections, see nature.com/documents/nr-reporting-summary-flat.pdf

Life sciences study design

All studies must disclose on these points even when the disclosure is negative.

Sample size	For crystal structures, 95 to 100% complete data sets were collected and used (Supplementary Table 1). For two cryo-EM structures, final sets of 146K and 157K single particles were used (Supplementary Table 4). Final sample size was determined when the quality of density maps were clearly resolved side for side-chains, and FSC resolution and R-free for cryo-EM and crystal structures, respectively, reached lowest values. HIV-RT RT inhibition assay contained sample size of n=3 biological replicates.
Data exclusions	No data excluded. X-ray and single particle cryo-EM data were processed using standard software packages.
Replication	HIV-1 RT inhibition assay (Fig. 5g) experiments were triplicated reproducibly. X-ray and cryo-EM datasets contained large sample sizes with final maps clearly resolving side chain density and therefore as a standard practice no replication of structure determination is required.
Randomization	As a standard practice, a random set of 5% data was used as R-free set in the refinement of each crystal structure to avoid model bias. Each cryo-EM structures was processed from large number of particles using standard protocol and software.
Blinding	As no animal or human subjects were involved, blinding was not relevant to our study. Reported data and findings were obtained from biophysical experiments where no blinding is required.

Reporting for specific materials, systems and methods

We require information from authors about some types of materials, experimental systems and methods used in many studies. Here, indicate whether each material, system or method listed is relevant to your study. If you are not sure if a list item applies to your research, read the appropriate section before selecting a response.

Materials & experimental systems

n/a	Involved in the study	
x		Antibodies
x		Eukaryotic cell lines
x		Palaeontology and archaeology
x		Animals and other organisms
x		Human research participants
x		Clinical data
x		Dual use research of concern

Methods

n/a	Involved in the study	
x		ChIP-seq
x		Flow cytometry
x		MRI-based neuroimaging
